# Single-Cell Transcriptome Reveals the Regulatory Role of STAT3 in Diquat-Induced Oxidative Stress in Piglet Hepatocytes

**DOI:** 10.3390/ijms26189161

**Published:** 2025-09-19

**Authors:** Yunpeng Li, Jia Li, Hongjin Li, Chu Zhang, Yongqing Zeng, Jin Wang, Wei Chen

**Affiliations:** Shandong Provincial Key Laboratory for Livestock Germplasm Innovation & Utilization, College of Animal Science and Technology, Shandong Agricultural University, Tai’an 271017, China; m17634803071_1@163.com (Y.L.); 15315491443@163.com (J.L.); hongjinli7831@163.com (H.L.); zhangchu1229@163.com (C.Z.); yqzeng@sdau.edu.cn (Y.Z.); 15254817801@163.com (J.W.)

**Keywords:** diquat, macrophage, oxidative stress, *STAT3*, liver

## Abstract

Oxidative stress (OS) is known to cause severe liver injury in weaning piglets; however, the cellular and molecular mechanisms underlying this process remain poorly understood. In this study, we employed a diquat (DQ)-induced OS model in weanling piglets and performed single-cell transcriptome sequencing of liver tissue to elucidate the key molecular and cellular events involved in OS-induced hepatic damage. First, piglets were treated with 12 mg/kg DQ and the same amount of saline, and the histopathology, biochemical indicators, and single-cell RNA sequencing (scRNA-seq) of piglets were analyzed. Mouse hepatocytes were used to verify the mechanism of differentially expressed genes, including *STAT3* knockdown/overexpression, reactive oxygen species (ROS) detection and apoptosis assay. DQ exposure caused significant oxidative damage in the liver of piglets, which was manifested as decreased superoxide dismutase (SOD) activity (*p* < 0.05), glutathione (GSH) consumption (*p* < 0.05) and increased malondialdehyde (MDA) (*p* < 0.05). Cell type-specific responses were revealed by scRNA-seq, with hepatocytes showing the most pronounced transcriptomic alterations (752 genes up-regulated and 918 genes down-regulated). The expression of *STAT3* was up-regulated in hepatocytes (*p* < 0.05) and down-regulated in B cells. The functional enrichment of macrophages involved FOXO/MAPK signaling and OS pathways. In vitro experiments showed that DQ treatment (IC50 = 125.8 μmol/L) led to an increase in ROS content and apoptosis, *STAT3* silencing aggravated ROS and apoptosis (*p* < 0.05), and *STAT3* overexpression alleviated ROS and apoptosis (*p* < 0.05). STAT3 activation increases HO-1 and Bcl-2, while inhibiting Bax and shifting the Bax/Bcl-2 ratio toward cell survival. It has been shown that DQ induces OS and apoptosis in a cell type-dependent manner, in which *STAT3* plays a key regulatory role in antioxidant defense and cell survival. Targeting *STAT3* may be a therapeutic strategy for DQ-induced hepatotoxicity.

## 1. Introduction

Oxidative Stress (OS) is defined as an imbalance of the body’s protective mechanisms caused by oxidation-reduction imbalance [1]. This imbalance can damage cellular macromolecules [2], cause OS damage and cell death, and cause cell death. This may indirectly affect the function and survival of the whole organism [3].

The liver is the main target organ for reactive oxygen species (ROS) attack [4] and the largest detoxification organ in the body. It is the most cutting-edge immune organ in the body [5]. Liver tissue contains a large number of mitochondria, which are the main source of ROS, so when OS occurs, the liver becomes the main organ of ROS attack [6]. The proteins, lipids and DNA of hepatocytes are mainly affected by ROS [7], which is the main reason for the occurrence of liver damage caused by OS and the development of severe liver diseases [8].

Diquat (DQ) is a widely used non-selective herbicide. As with other approved plant protection products, its application is subject to strict regulations regarding dosage and pre-harvest intervals—ranging from 7 days for certain fruits and vegetables to 14 days for potatoes. It is important to note that the toxic effects discussed in this study may arise not only from excessive application exceeding recommended thresholds, but also from potential misuse such as failure to observe waiting periods or incorrect dosing, even when initial application follows guidelines. Although DQ is being phased out in some regions such as the European Union in favor of natural alternatives like pelargonic acid, it remains in use in many areas [9].

In livestock production, DQ residues may persist in forage crops or forage, posing a risk of exposure to animals. This is particularly concerning in weaned piglets, which represent a critical growth stage in pig production. DQ can utilize molecular oxygen to generate O_2_^−^, which is subsequently converted to hydroperoxide. These reactive species induce lipid peroxidation of cell membranes, ultimately leading to apoptosis. Studies have reported that the primary organ affected by DQ toxicity is the liver [10]. In weaned piglets, DQ-induced OS significantly compromises growth performance and may increase susceptibility to other diseases later in life, thereby severely impacting production efficiency [11]. Moreover, DQ poses health risks to humans, including kidney damage, irritation of the mucous membranes in the throat, esophagus, and stomach, as well as potential injury to human hepatocytes through similar mechanisms of OS.

In the regulation of liver injury, *STAT3* as a key transcription factor in the JAK-STAT signaling pathway, plays an important role in promoting cell survival and proliferation by recognizing phosphorylated tyrosine through its SH2 domain [12]. However, persistent activation of *STAT3* may have deleterious effects [13], leading to multiple pathological states [14]. This dual protective and pathogenic role makes STAT3 a key factor in determining cell fate under oxidative conditions. Therefore, exploring the regulatory mechanism of *STAT3* in DQ-induced hepatotoxicity may provide new insights into elucidating its toxicological effects and developing intervention strategies.

A previous transcriptomic analysis conducted in a piglet model of OS induced by intraperitoneal injection of DQ revealed widespread changes in gene expression [15]. To further decipher cell type-specific responses and better understand the heterogeneity of hepatic reactions, we applied 10× single-cell RNA sequencing. This approach allows for the identification of differentially expressed genes across distinct cell populations, offering a comprehensive perspective on how DQ affects liver function, immune regulation, and antioxidant capacity in piglets. Weaned piglets were selected as a representative livestock species due to their metabolic similarity to humans, high sensitivity to OS, and relevance in agricultural toxicology research. This model provides a translational platform for investigating oxidative injury mechanisms and evaluating potential therapeutic strategies aimed at mitigating chemical-induced hepatotoxicity.

## 2. Results

### 2.1. Detection of DQ-Induced Liver Histopathology and OS-Related Traits

**Figure 1:**
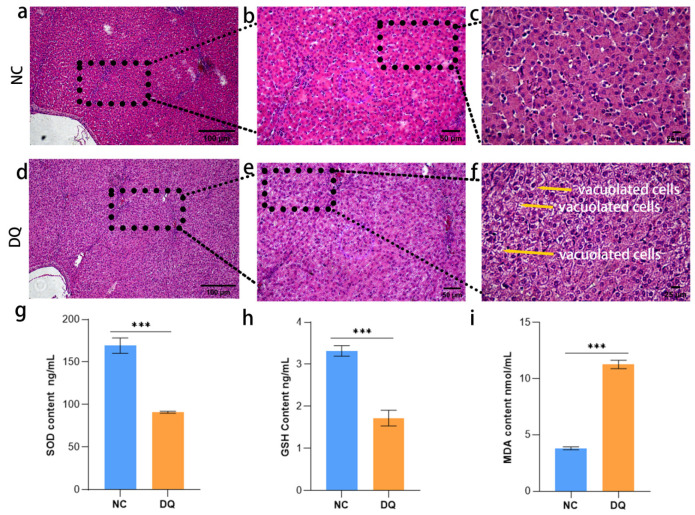
Assessment of liver histopathology with OS-related SOD, MDA, and GSH levels. (**a**–**f**) Representative hematoxylin and eosin (H&E) stained tissue sections at different magnifications: (**a**–**c**) negative control groups (using an equal volume of saline), and black boxes in (**a**) indicate enlarged areas in (**b**,**c**). (**d**–**f**) DQ treated group, showing extensive inflammatory infiltration (yellow arrows) and vacuolated cells (white text), suggesting cell damage. Scale bars: 100 μm (**a**,**d**), 50 μm (**b**,**e**), and 25 μm (**c**,**f**). (**g**) superoxide dismutase (SOD); (**h**) Reduced glutathione (GSH); (**i**) malondialdehyde (MDA). Values are presented as mean ± SD. * p < 0.05, ** p < 0.01, *** p < 0.001.

Histopathological examination under light microscopy revealed no apparent inflammatory response in the negative control (NC) group. In contrast (Figure 1a–c), the DQ group exhibited significant inflammatory infiltration in hepatic tissues (10×−40×), with vacuolated cells dispersed among inflammatory cells at higher magnification (40×) (Figure 1d–f). OS analysis demonstrated that the DQ group had markedly reduced SOD activity compared to controls (*p* < 0.001), accompanied by significantly decreased GSH levels (*p* < 0.001) and elevated MDA content (*p* < 0.001). These findings indicate that OS led to diminished antioxidant enzyme activity, depletion of GSH, and lipid peroxidation (Figure 1g–i).

### 2.2. Single-Cell RNA Sequencing Reveals the Characteristics of Porcine Liver Cell Types

**Figure 2:**
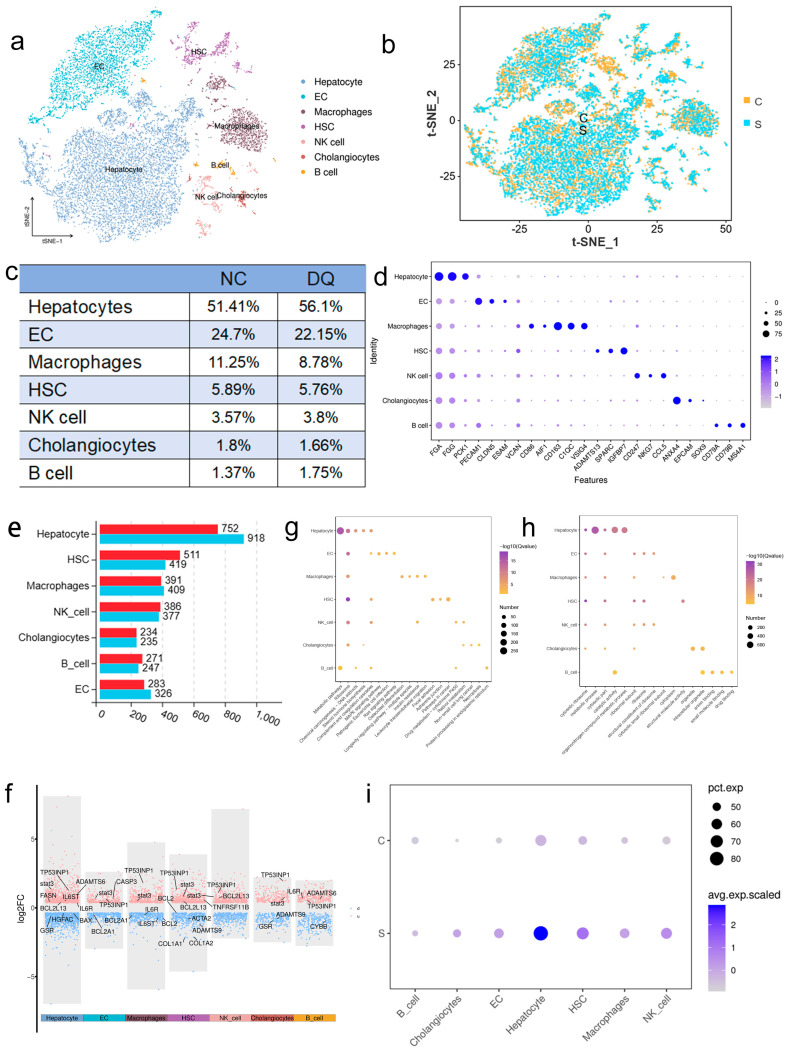
Analysis of single-cell transcriptomic features of the liver. (**a**) t-SNE visualization of liver subsets, demonstrating the heterogeneity of cell clusters. (**b**) Comparative t-SNE distributions between the two experimental groups, revealing similar proportional representation of the subgroups. (**c**) Component analysis of seven different subgroups for percentage-based statistics. (**d**) Bubble plot showing characteristic markers used for subgroup identification. Features (*X*-axis) are plotted against cell identity (*Y*-axis), and bubble sizes represent expression levels. (**e**) Bar graph showing differentially expressed genes in subgroups. Statistical significance was used (adjusted *p* < 0.05). (**f**) Volcano plot illustrating the expression patterns of OS-related genes in the seven subpopulations. Red and blue dots represent significantly up-regulated and down-regulated genes (adjusted *p* < 0.05, |log2 fold change |> 1), respectively. (**g**) KEGG pathway enrichment analysis. The top five significantly enriched pathways for each subgroup are shown, bubble sizes correspond to gene counts, and color intensities reflect −log10 (*p*-value). (**h**) GO enrichment analysis was divided into biological process (BP), cellular component (CC), and molecular function (MF). For each category, the top five most significantly enriched terms (−log10 (*p*-value)) will be shown. (**i**) Expression profiles of candidate gene *STAT3* in all subsets, visualized as bubble plots, where size corresponds to relative expression abundance.

To reveal the cellular features of liver injury in piglets, we administered 12 mg/kg DQ and saline (*n* = 1) intraperitoneally to 2 piglets (30 days of age) every 2 days for 7 days, after which single-cell RNA sequencing of liver tissue was performed, and after quality control, 9011 (NC) and 9040 (DQ) cells were retained. Semi-supervised clustering identified seven cell populations (Figure 2a): hepatocytes (51.41% vs. 56.1%), endothelial cells (24.7% vs. 22.15%), macrophages (11.25% vs. 8.78%), hepatic stellate cells (5.89% vs. 5.76%), and NK cells (3.57% vs. 3.8%), bile duct cells (1.8% vs. 1.66%), and B cells (1.37% vs. 1.75%), and the proportion of cells in the NC and DQ group was roughly similar (Figure 2b). DQ treatment induced a cell type-specific OS response: OS was significantly increased (4.69%) in hepatocytes, while it was decreased (−2.47%) in macrophages (Figure 2c). These differential responses highlight the different mechanistic roles of OS pathways during liver injury, providing insights for future studies in liver disease models.

Seven conserved cell types were identified in porcine liver by transcription factor marker gene analysis (Figure 2d): hepatocytes (*FGA*, *FGG*, *PCK1*), endothelial cells (*PECAM1*, *CLDN5*, *ESAM*), macrophages (*VCAN*, *CD68*, *AIF1*), hepatic stellate cells (*ADAMTS13*, *SPARC*, *IGFBP7*), NK cells (*CD247*, *NKG7*, *CCL5*), cholangiocytes (*ANXA4*, *EPCAM*, *SOX9*) and B cells (*CD79A*, *CD79B*, *MS4A1*). Differential expression analysis showed the most significant changes in gene expression in hepatocytes (752 up-regulated and 918 down-regulated) and balanced changes in macrophages (391/409) (Figure 2e). The volcano plot shows cell-specific expression of OS-related genes such as *TP53INP1* and *IL6R* (Figure 2f). KEGG pathway analysis showed that drug metabolism and insulin signaling pathways such as *IGFBP1* were significantly enriched (Figure 2g), and GO analysis revealed drug binding and metabolic regulation processes (FDR < 0.05) (Figure 2h).

KEGG/GO analysis suggested that FOXO1 pathway played a key role in DQ-induced OS model, and *STAT3* might be indirectly regulated by FOXO1 pathway. Single-cell analysis showed that *STAT3* was down-regulated in B cells (*p* < 0.05), but up-regulated in cholangiocytes, endothelial cells, hepatic stellate cells, macrophages and NK cells (*p* < 0.05), especially in hepatocytes (*p* < 0.05). We focused on the role of STAT3 in OS in hepatocytes, and sub-cluster analysis of macrophages was performed to explore the molecular mechanism.

### 2.3. Single-Cell RNA Sequencing Reveals Macrophage Subsets

**Figure 3:**
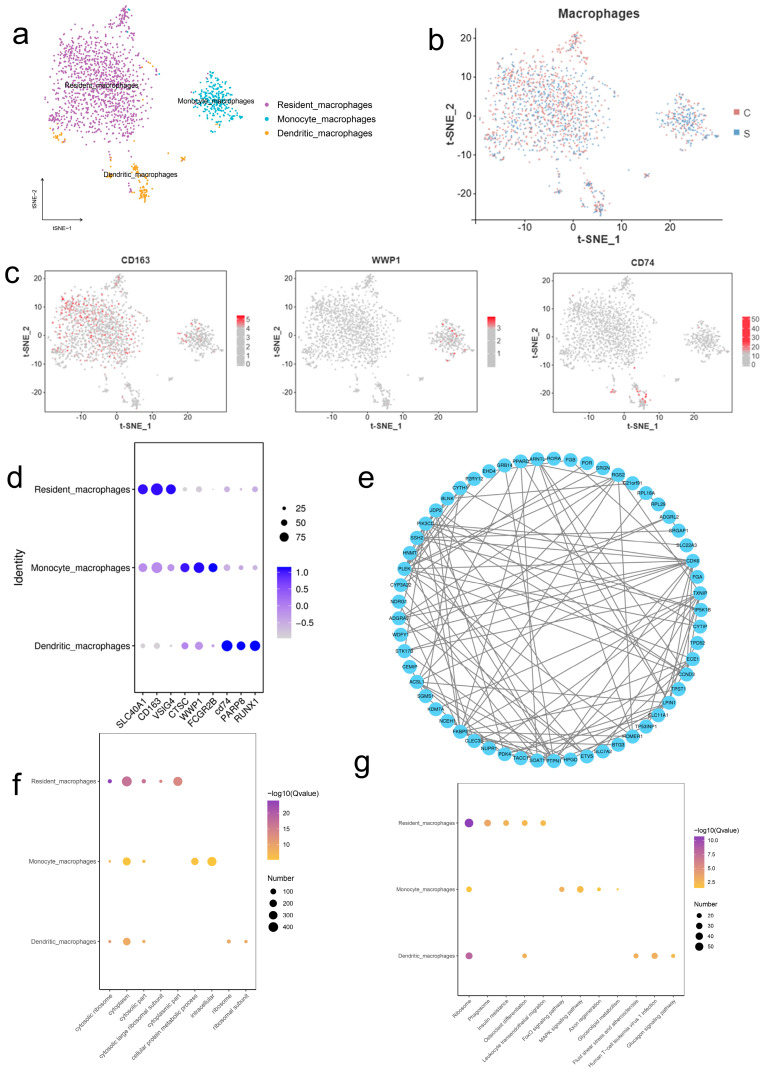
Single-cell transcriptomic and proteomic features of macrophage subsets. (**a**) t-SNE projections of macrophage clusters identified by single-cell RNA sequencing analysis, showing cellular heterogeneity among subpopulations. (**b**) Comparative analysis of the distribution of macrophage subsets in each experimental group (group C and group S). (**c**) Spatial expression patterns of representative marker genes in t-SNE coordinates. Gene expression levels were normalized and visualized using color intensity gradients. (**d**) Bubble plots showing characteristic markers of the identified macrophage subsets. Bubble size indicates the percentage of cells expressing each marker, whereas the color scale indicates the mean expression level (log-normalized counts). (**e**) Protein–protein interaction (PPI) network of DEGs between macrophage groups (fold change > 2, *p* < 0.01). (**f**) GO enrichment analysis of differentially expressed proteins, showing the top five significantly enriched terms in each category: biological process (BP), molecular function (MF), and cellular component (CC). Sorted by (−log10 (*p*-value)). (**g**) KEGG pathway enrichment analysis of differentially expressed proteins, showing the top five most enriched pathways (−log10 (*p*-value)). The bubble size was plotted against the number of genes.

By semi-supervised clustering, macrophages were divided into three subpopulations: resident macrophages (*SLC40A1*, *CD163*, *VSIG4*), dendritic cells (*CD74*, *PARP8*, *RUNX1*), and mononocytic macrophages (*CTSC*, *WWP1*, *FCGR2B*) (Figure 3a–c). t-SNE analysis revealed a specific distribution of marker genes (Figure 3d). Differential analysis (logFC > 2, *p* < 0.01) identified 75 OS-related proteins (e.g., TP53INP1, TPST1), and the protein interaction network suggested reprogramming of signaling pathways induced by OS (Figure 3e). KEGG pathway analysis showed that FOXO and MAPK pathways were significantly enriched (Figure 3g) and GO analysis revealed that the ribosome biosynthesis process was up-regulated (FDR < 0.05), indicating that macrophages were key effector cells of OS (Figure 3f).

### 2.4. Establishment of OS Model in NCTC1469 Cells

**Figure 4:**
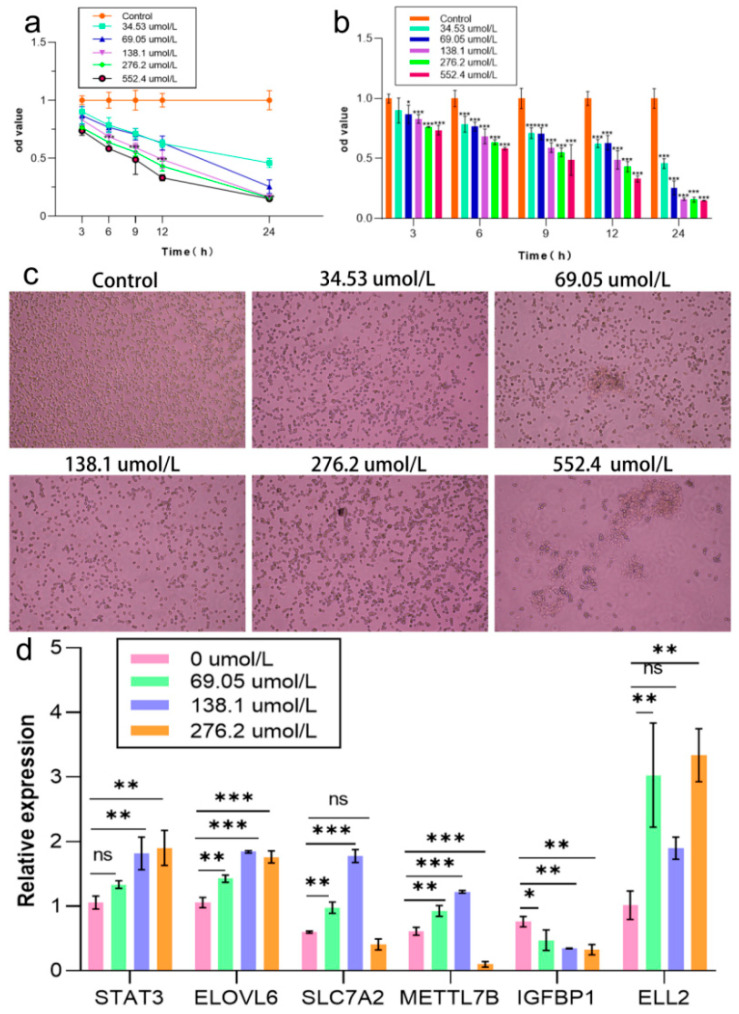
Dose- and time-dependent effects on apoptosis in the cell models. (**a**) Temporal apoptosis dynamics at fixed concentrations (*n* = 6 technological replicates). (**b**) Dose–response apoptosis profiles at fixed time points in a concentration-dependent apoptotic effect after exposure to different times. (**c**) Morphological characterization of cell state Phase contrast microscopy images (4×) showing different cell morphologies (normal/apoptotic/necrotic) after 12 h of treatment. (**d**) Molecular validation of cell models to establish RT-qPCR validation of DGEs in the models. The mRNA levels of genes were normalized to β-actin and expressed as fold change relative to control (2^−ΔΔCt^ method). Bar graphs represent mean ± SD (*n* = 3). * p < 0.05, ** p < 0.01, *** p < 0.001, ns = not significant.

The CCK-8 assay revealed the dose- and time-dependent cytotoxic effects of DQ on cells. At 12 h, with the increase in DQ concentration (34.53–552.4 μmol/L), the cell viability was significantly decreased, which was 100% ± 5.87% (control) and 33.03% ± 2.6% (552.4 μmol/L; *p* < 0.001) (Figure 4b) with an IC50 of 125.8 μmol/L. 138.1 μmol/L time course analysis (0–24 h) showed progressive loss of viability (100% ± 5.87% to 33.03% ± 2.6%; *p* < 0.05) (Figure 4a). Morphological changes were concentration-dependent (Figure 4c). Gene-expression analysis revealed significant alterations in *STAT3*, *ELOVL6*, *SCL7A2*, *METTL7B*, *IGFBP1*, and *ELL2* (*p* < 0.05 to 0.001) (Figure 4d). Based on these findings, 138.1 μmol/L (12 h, ~50% viability) was selected for subsequent experiments.

### 2.5. Annexin V/PI Dual-Staining Flow Cytometry Demonstrates the Role of STAT3

**Figure 5:**
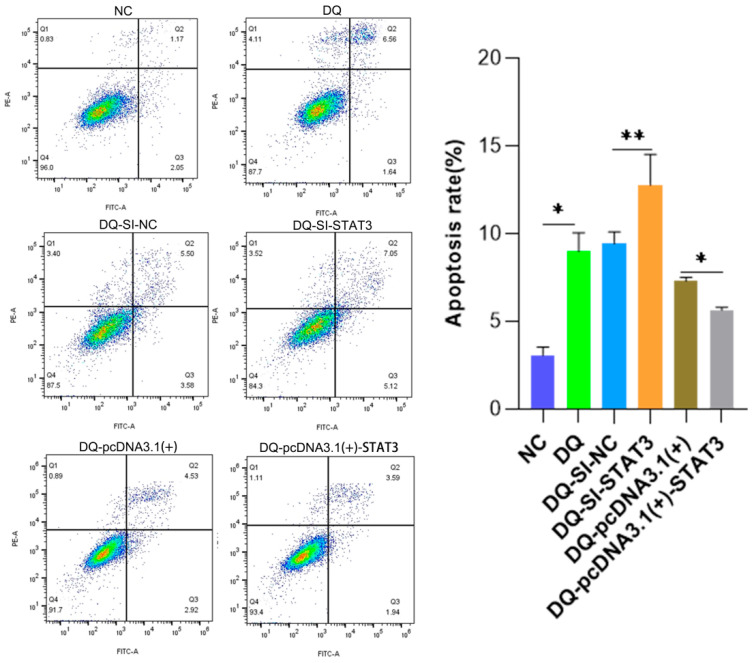
Effects of DQ treatment and DQ interference/overexpression of *STAT3* on apoptosis. * p < 0.05, ** p < 0.01, *** p < 0.001.

The apoptosis rate of the DQ group was significantly higher than that of the NC group (*p* < 0.001), and experimental treatment of DQ could effectively induce apoptosis (Figure 5). DQ-SI-STAT3 group vs. DQ-SI-NC group after *STAT3* knockdown (DQ-SI-STAT3), the apoptosis rate was further significantly increased (*p* < 0.001 vs. DQ-SI-NC), indicating that *STAT3* had an inhibitory effect on apoptosis, and its deletion would aggravate apoptosis. DQ-pcDNA3.1(+) vs. DQ-pcDNA3.1(+)-STAT3; The apoptosis rate of *STAT3* overexpression (DQ-pcDNA3.1(+)-STAT3) was significantly lower than that of empty vector control group (*p* < 0.01 vs. DQ-pcDNA3.1(+)), which further verified the anti-apoptosis function of *STAT3*.

### 2.6. Fluorescence Microscopy Analysis of ROS in Cells

**Figure 6:**
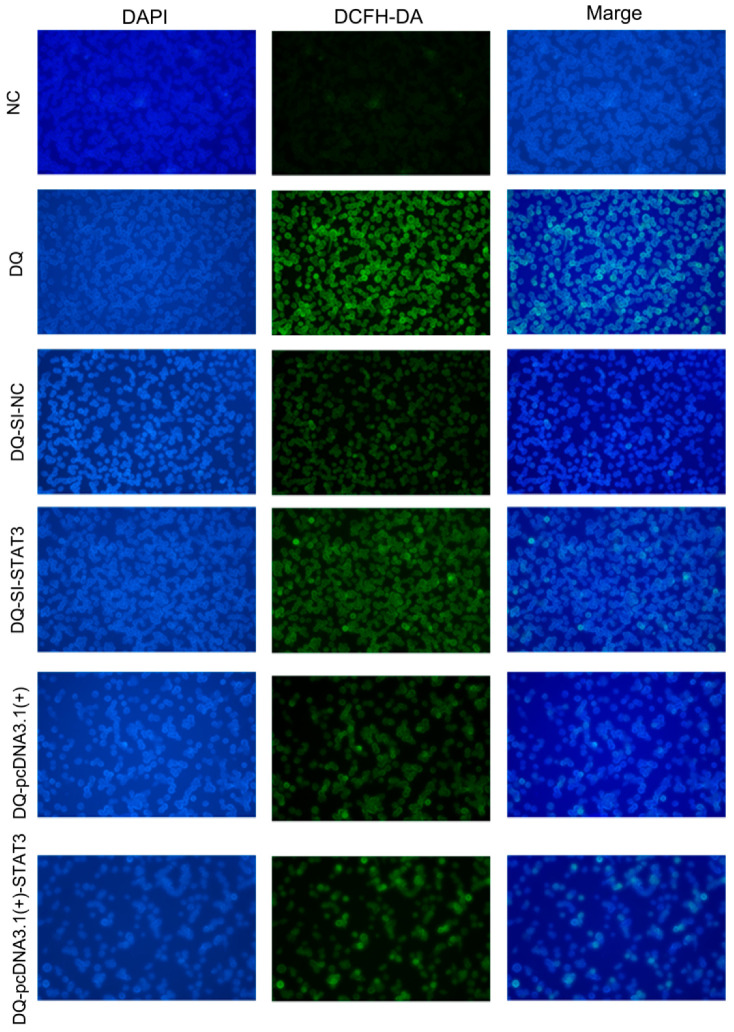
Intracellular ROS detection. From left to right (20×): DAPI nuclear staining (blue), DCFH-DA detection of ROS (green), and merged images showing colocalization.

Representative fluorescence microscopy of ROS levels detected by DCFH-DA staining (Figure 6). The DQ group exhibited significantly enhanced green fluorescence intensity compared to the NC group, indicating increased ROS accumulation. Notably, *STAT3* knockdown (DQ-SI-STAT3) further increased ROS levels compared with negative control SIRNA (DQ-SI-NC), However, *STAT3* overexpression (DQ-pcDNA3.1(+)-STAT3) reduced ROS intensity relative to the empty vector control (DQ-pcDNA3.1(+)). These findings suggest that *STAT3* plays an inhibitory role in ROS generation.

### 2.7. Effects of Cellular Antioxidant Genes and Key Genes of Apoptosis

**Figure 7:**
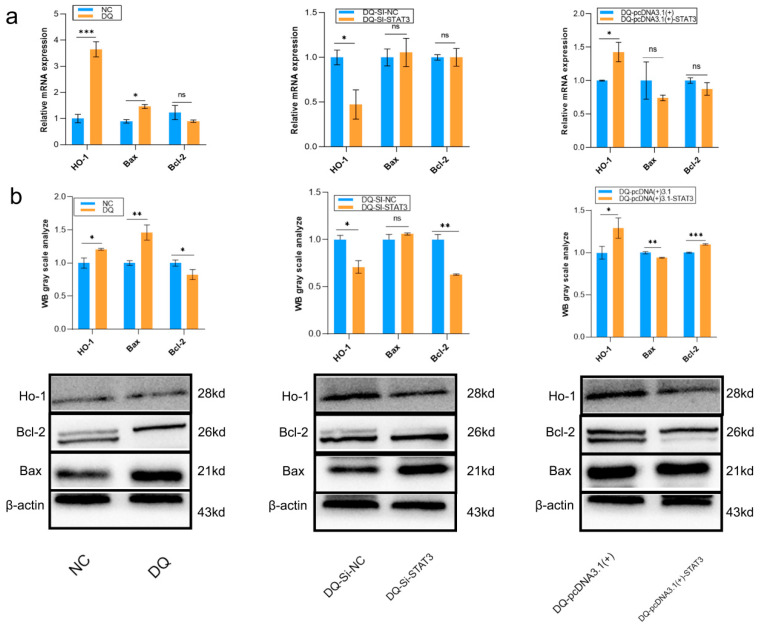
Detection of HO-1, Bax and Bcl-2 expression. (**a**) Relative mRNA expression levels of *HO-1, Bax*, and *Bcl-2* as determined by real-time quantitative PCR (RT-qPCR). Data were normalized to *β-actin* and presented as mean ±SD (*n* = 3). (**b**) Representative Western blot analysis of HO-1, Bax, and Bcl-2 protein expression. Top: Grayscale quantification of protein bands, expressed as the relative density normalized for β-actin. Bottom: Original immunoblot images showing different protein bands corresponding to each target. * p < 0.05, ** p < 0.01, *** p < 0.001, ns = not significant.

The role of STAT3 was initially assessed in Supplementary Material S1, where it was found to play a dual regulatory role in modulating both HO-1 expression and the Bax/Bcl-2 balance under DQ-induced OS. Compared to the control group, DQ treatment significantly increased the mRNA and protein levels of HO-1 and the pro-apoptotic protein Bax, while decreasing the expression of the anti-apoptotic protein Bcl-2 (Figure 7a). This resulted in a marked elevation of the Bax/Bcl-2 ratio (>1), indicating a shift toward apoptosis.

STAT3 silencing (DQ-SI-STAT3) further suppressed HO-1 expression but did not significantly affect the Bax/Bcl-2 ratio. In contrast, STAT3 overexpression (DQ-pcDNA3.1(+)) enhanced the expression of both HO-1 and Bcl-2, while reducing Bax levels, thereby lowering the Bax/Bcl-2 ratio and attenuating apoptosis (Figure 7b). Protein-level analyses corroborated these trends, confirming that STAT3 activation exerts an anti-apoptotic effect by promoting antioxidant response via HO-1 and shifting the Bax/Bcl-2 balance toward cell survival.

Together, these results demonstrate that STAT3 mitigates DQ-induced cytotoxicity by concurrently alleviating OS (through up-regulation of HO-1) and inhibiting apoptosis (via restoration of the Bax/Bcl-2 balance). These findings underscore the potential of STAT3 as a therapeutic target in OS-related diseases.

**Figure 8:**
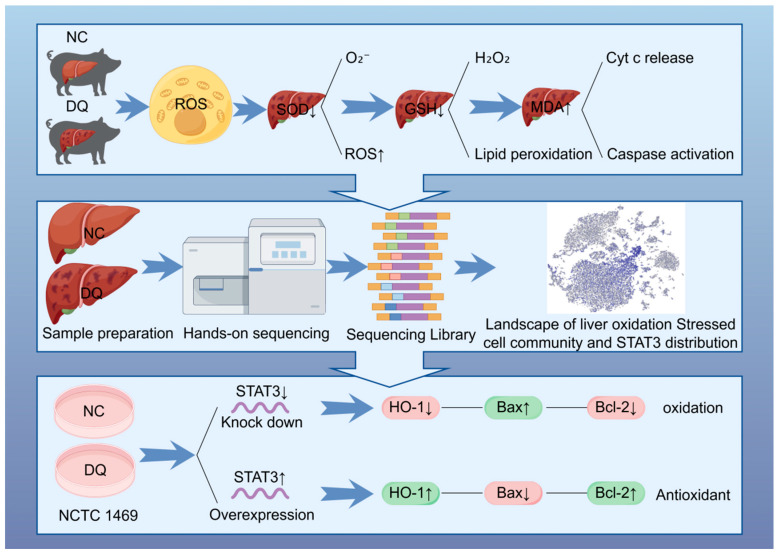
Analysis of the regulatory network of *STAT3* on OS in pig liver based on single-cell sequencing.

In this study, single-cell RNA sequencing was used to analyze cell phenotypes and assess OS markers, including SOD, GSH, and MDA. *STAT3* was identified as a key regulator by differential gene expression analysis. To further validate its role, a cell model was established using the mouse liver cell line NCTC 1469 under OS induced by DQ. The expression levels of apoptosis-related genes (*Bcl-2*, *Bax*) and antioxidant gene *HO-1* were detected. The results showed that *STAT3* played an anti-apoptotic role in DQ-induced OS, highlighting its critical role in mitigating cell damage (Figure 8).

## 3. Discussion

This study integrated histopathology, biochemistry, and single-cell transcriptomic analyses to provide comprehensive insights into the mechanism of DQ-induced liver injury. Our findings suggest that DQ exposure induces significant OS [16], inflammation, and apoptosis in liver tissue, and that *STAT3* plays the key regulatory role in these processes [17].

Histopathological examination revealed significant inflammatory infiltration and vacuolar cells in DQ-treated liver tissue, consistent with previous reports on herbicidine-induced liver injury. The significant decrease in SOD activity and GSH levels, coupled with the increase in MDA, confirmed severe oxidative damage, which is consistent with the production of ROS by DQ via REDOX cycling. These biochemical alterations were further confirmed by single-cell RNA sequencing (scRNA-seq), which revealed the differential expression of OS-related genes (such as *TP53INP1*, *IL6R*, *ADAMTS6*) in liver cell populations. Notably, the enrichment of FOXO and MAPK signaling pathways in macrophages suggests their critical role in mediating OS responses, as these pathways are known regulators of antioxidant defense and inflammation [18].

scRNA-seq analysis also uncovered distinct transcriptional profiles among different liver cell types. Hepatocytes showed the most substantial changes in gene expression, indicating their high metabolic susceptibility to DQ [19]. The conserved expression of cell-specific markers such as *FGA* in hepatocytes and *PECAM1* in endothelial cells across species reinforces the translational relevance of our porcine model [20]. Additionally, the differential expression of *IGFBP1*—a known anti-apoptotic factor [21]—in hepatocytes further supports its potential protective role against DQ-induced toxicity [22].

A key finding of our scRNA-seq data is the cell type-specific dysregulation of *STAT3*, which was significantly up-regulated in hepatocytes but down-regulated in B cells. *STAT3* has been implicated in anti-apoptotic responses across various liver injury models [23]. Consistent with this, *STAT3* silencing has been shown to exacerbate apoptosis and ROS accumulation [24], whereas its overexpression can attenuate these effects through modulation of the *HO-1* and *Bax/Bcl-2* pathways. The up-regulation of *HO-1*—a cytoprotective enzyme—in STAT3-overexpressing cells further highlights its contribution to antioxidant defense [25]. Conversely, *STAT3* knockdown increased the Bax/Bcl-2 ratio and promoted apoptosis [26]. These results are in line with prior studies suggesting that STAT3 contributes to adaptive responses against OS [27], although its pleiotropic and context-dependent functions warrant further investigation [28].

Beyond its well-established role in inflammation and cell survival, *STAT3* has emerging therapeutic potential. Targeting *STAT3* signaling—either through activation or inhibition—represents a promising strategy for modulating OS and apoptosis in chemical-induced liver injury. For instance, small-molecule activators of *STAT3* may confer hepatoprotection in DQ poisoning by enhancing antioxidant gene expression and suppressing pro-apoptotic pathways.

Although our study clarifies the role of *STAT3* in DQ hepatotoxicity, the small sample size may limit generalizations [29]. Future studies should validate these findings in larger cohorts and explore the interaction of *STAT3* with other signaling pathways such as Bcl-2, Nrf2 [30]. In addition, in vivo mechanistic studies are needed to evaluate the regulatory role of therapeutic *STAT3* in DQ poisoning [31].

## 4. Materials and Methods

This animal study was reviewed and approved by the Animal Ethics Committee of Shandong Agricultural University and was conducted in accordance with the guidelines and regulations of the committee (SDAUA-2021-041).

### 4.1. Experimental Animals and Cells

Six weaned piglets (30 days old) from Zaozhuang Heigai pig were raised in the same environment. The OS group was injected with 12 mg/kg DQ, and the control group was injected with the same amount of normal saline. Two pieces of liver tissue of each pig were collected, one was placed in a frozen tube, and the frozen tube was placed in a liquid tank for 10× single-cell sequencing, and the other was placed in a 50 mL centrifuge tube, and 4% paraformaldehyde was added to fix the tissue for histopathological study.

Mouse liver cells (NCTC1469) were used to establish the cell model. The cells were from Saibaikang (Shanghai, China) Biotechnology, and the model concentration was calculated from the laboratory’s pre-production data [15].

### 4.2. Hematoxylin and Eosin (HE) Staining

Fresh liver tissue from Zaozhuang Heigai pig was collected and fixed with 4% formaldehyde solution for more than 24 h. The fixed tissue was dehydrated in an AS dehydration apparatus, followed by tissue embedding using a HistoSTAR embedding machine, and then the waxed tissue blocks were cut into 5 µm thick sections using a Microm HM 355S microtome. They were stained with hematoxylin and eosin, placed in a 37 °C drying box overnight, and observed by microscope when water vapor had completely disappeared.

SOD, MDA, and GSH levels in liver tissues. For SOD, GSH and MDA detection, tissue samples: About 0.1 g of tissue (0.25 g for well-hydrated samples) was added to 1 mL of the corresponding extract and homogenized at 4 °C or in an ice bath. The samples were centrifuged at 4 °C × 12,000 rpm for 10 min, and the supernatant was used as the solution to be tested (Supplementary Material S2).

### 4.3. Single-Cell RNA Sequencing

We performed 10× single-cell RNA sequencing on fresh porcine liver samples (saline C and DQ S, *n* = 1) using the Chromium Next GEM Single Cell 3’ Kit v3.1(10× Genomics, Pleasanton, CA, USA). Libraries were sequenced on an Illumina HiSeq 4000. Data were processed using Cell Ranger (v3.1.0) aligned with *Sus scrofa* 11.1. Quality control in Seurat (v3.1.1) consisted of removing DoubletFinder and filtering cells with 500 to 4000 genes >8000 UMI, or >10% mitochondrial genes. Expression was normalized using log normalization.

The formula is given as follows.

$$A\;\mathrm{gene}\;\mathrm{expression}\;\mathrm{level}=\log\;(1\;+\;\frac{\mathrm{UMIA}}{\mathrm{UMITotal}}\times 10000)$$

After Harmony-based batch correction, PCA was performed to select the best PC for clustering (Seurat) and t-SNE visualization. The published tags were used for cell annotation. Scanpy’s rank-sum test identified cluster-specific degs (|log2FC| > 0.36, *p* < 0.01, >25% cells) and between-group degs (|log2FC| > 0.36, *p* < 0.05). GO/KEGG enrichment (Omicsmart) employs hypergeometric testing in a genomic context.

The formula is given as follows.

P=1−∑i=0m−1MiN−Mn−iNn

The number of genes in N, where *n* is the number of DEGs. M is the number of all genes annotated with a particular GO term, or KEGG pathway, and m is the number of DEGs in M. The calculated *p* values were FDR corrected, and FDR ≤ 0.05 was used as the threshold. GO terms fulfilling this condition were defined as GO terms that were significantly enriched in DEGs. This analysis was able to identify the major biological functions of the DEGs.

The volcano plot is log2FC on the *X*-axis and -log10 on the *Y*-axis (*P-*adj). The red dots (|log2FC|> 0.36, *p* <0.05) show the up-regulated genes of Cluster A, and the blue dots show the down-regulated genes. The horizontal axis of the bubble plot shows GO terms (such as ribosomes, metabolic processes, etc.) and the vertical axis shows cell types. Bubble color reflects enrichment significance (-log10(Q-value), purple most significant, and size represents gene/sample number.

STRING (confidence ≥ 0.7) was used to analyze porcine macrophage DEGs, and then Cytoscape software, version 3.9.1-based PPI network construction, topological parameter calculation and key node/module identification were performed to elucidate functional interactions.

### 4.4. Cell Culture and Cell Model Establishment

NCTC1469 cell line was cultured in high-glucose Dulbecco’s modified Eagle’s medium (Gibco, Grand Island, NY, USA) supplemented with 10% horse serum (Gibco, NY, USA). and 0.5% penicillin/streptomycin (Solarbio, Beijing, China) were incubated at 37 °C in a humidified atmosphere containing 5% CO_2_.

The cell model was established by mixing medium and DQ, and the cells were grown to 90% density cells. The medium containing DQ was replaced, and the concentration of DQ was (0 µmol/L, 34.53 µmol/L, 69.05 µmol/L, 138.1 µmol/L, 276.2 µmol/L, 552.4 µmol/L, respectively), the incubation time was (0 h, 3 h, 6 h, 9 h, 12 h, 24 h), and the concentration was calculated from the previous production data of the laboratory [15].

### 4.5. Cell Viability Assay

The viability of NCTC1469 cells was determined by CCK-8 assay. Cells were seeded in 96-well plates and cultured to 90% density. Before the experiment, the normal cultured NCTC1469 cells were taken, the original culture medium was removed, washed with PBS, and digested with trypsin for 2 min. Then the digestion was terminated, and the cells were blown into single cells. The remaining cell suspension was centrifuged at 1000 rpm for 5 min, resuspended in medium, plated at 10,000 cells/well (96-well plates), and treated with DQ after overnight attachment.

### 4.6. Cell Transfection

The expression vector was transfected into NCTC1469 using Lipofectamine 2000 (Thermo Fisher Scientific, Waltham, MA, USA). Cells were seeded in 6-well cell culture plates before transfection. Transfection was performed when the cells were 90% grown. For the preparation of SIRNA transfection complex (dose per well), liquid A: 100 μL Opti-MEM (Gibco, USA) + 8 μL siRNA. Liquid B: 100 μL Opti-MEM + 5 μL Lipofectamin2000 reagent. For preparation of STAT3-pcDNA3.1 transfection complex, liquid A: 100 μL Opti-MEM + 4 ug of overexpression plasmid; Liquid B: 100 μL Opti-MEM + 5 μL Lipofectamin2000 reagent. After that, the culture plates were kept at room temperature for 5 min before liquid B was added to liquid A and left for 20 min. 1 mL of prewarmed Opti-MEM was administered to each cell well. A total of 200 μL of the prepared mixture was added to the cells in each well and mixed gently. After 6 h of incubation, the complete medium was replaced.

### 4.7. Total RNA Extraction, cDNA Synthesis, and Real-Time RT-PCR

The RNA extraction kit as well as the reverse transcription kit and the SYBR Green Pro Taq HS premixed qPCR kit were from Accurate Biotechnology (AG, Changsha, Hunan, China). RNA extraction and reverse transcription were performed on ice to maintain RNA integrity. In brief, 1 μg of total RNA in 20 μL reaction mixture reverse transcriptase was used to synthesize first-strand cDNA following the following procedure: 15 min at 37 °C, 5 s at 85 °C, and storage at 4 °C. Gene expression was analyzed using cDNA (2 μL) and gene-specific primers 10× (0.4 μL F, 0.4 μL R), SYBR Premix Ex Taq(2x) (10 μL), and free Water 7.2 μL. All reactions were performed in triplicate, and SPSS software, version 25.0 was used to calculate the relative amount of normalized gene expression versus control ^®^−∆Ct, where ∆Ct = Ct gene − Ct control [32]. *β-actin* was used as an internal control.

### 4.8. Cellular ROS Activity Assay

To measure intracellular ROS, cells on cover slides of 6-well plates were rinsed with precooled PBS and then incubated with 1 mL of diluted DCFH-DA (Beyotime Biotechnology, Shanghai, China) for 20 min at 37 °C (mixing every 3 min). After washing with serum-free medium, coverslips were mounted on slides containing DAPI with an anti-quench mounting agent, sealed with nail polish, and imaged at 20× magnification using a fluorescence microscope.

### 4.9. Detection of Apoptosis

The treated cells were centrifuged at 1300 rpm for 5 min, the supernatant was discarded, the cells were collected, and the cells were washed twice with PBS. Fifty to 100,000 cells were collected, and the cells were gently resuspended by adding 195 μL of Annexin V-FITC binding solution. 5 μL Annexin V-FITC and 10 μL propidium iodide staining solution were added and mixed gently. The cells were incubated at room temperature (20–25 °C) in the dark for 10–20 min and then detected by the machine.

### 4.10. Western Blot Analysis

Cell samples were collected, homogenized in 1:10 (*w*/*v*) cold lysis buffer, and then centrifuged at 12,000× *g* at 4 °C for 5 min to obtain the supernatant. Protein concentration was quantified using the BCA protein assay kit (P0010, Beyotime biotechnology, Shanghai, China). About 10 μg of protein samples were separated by 10% SDS-PAGE gel electrophoresis and transferred to a polyvinylidene fluoride (PVDF) membrane at 200 mA for 2 h. The membrane was then blocked with 5% skimmed milk powder at room temperature for 2 h, followed by an overnight incubation with the primary antibody at 4 °C. The antibodies used were as follows: HO-1 antibody, Bax antibody, Bcl-2 antibody, which were washed three times with PBST and incubated with the secondary antibody at room temperature for 2 h. Protein bands were visualized using ECL reagent and the signal intensity was analyzed using Quantity One software, version 7.2 (Bio-Rad, Hercules, CA, USA).

### 4.11. Statistic Analysis

All data were analyzed using IBM SPSS Statistics 20 software, and the statistical significance of differences between groups was checked using Student’s *t*-test. Images were plotted using GraphPad Prism 5.0, and data for each group are expressed as mean ± SD. Statistical significance was expressed as * p < 0.05, ** p < 0.01, *** p < 0.001.

## 5. Conclusions

In summary, our single-cell transcriptome approach revealed that DQ induces OS, inflammation, and apoptosis in the liver, revealing transcriptomic alterations evident mainly in the hepatocyte and macrophage populations. Analysis of differences between groups identified FOXO1-mediated regulatory mechanisms as the main driver of these cell type-specific differences, accompanied by the activation of OS, inflammatory cascades, and apoptotic pathways. *STAT3* is a key regulator of these processes. *STAT3* activation provides cytoprotection by enhancing *HO-1* expression and restoring *Bax/Bcl-2* balance, whereas its inhibition exacerbates ROS-mediated injury. These findings position *STAT3* as a potential therapeutic target to mitigate DQ-induced hepatotoxicity, providing a new approach for the treatment of OS-related liver injury. Further studies should focus on translational applications, including *STAT3*-targeted therapy for herbicide poisoning.

## Data Availability

The original contributions presented in the study are included in the article; further inquiries can be directed to the corresponding authors.

## Supplementary Materials

The following supporting information can be downloaded at: https://www.mdpi.com/article/10.3390/ijms26189161/s1.
