# Supplementary material for: Single-Cell Transcriptome Reveals the Regulatory Role of STAT3 in Diquat-Induced Oxidative Stress in Piglet Hepatocytes
Source: Int J Mol Sci. 2025 Sep 19;26(18):9161. doi: 10.3390/ijms26189161 (PMC12471023; doi:10.3390/ijms26189161)
Supplement: Supplementary file 1 [file ijms-26-09161-s001.zip › Supplementary Material S1.pdf]

Validation of STAT3 knockdown/overexpression efficiency in NCTC 1469 cells

The stat3-mus-690 interference group exhibited the highest interference efficiency, showing a significant reduction compared to the control group ($P < 0.001$). The stat3-mus-1531 interference group also demonstrated high interference efficiency ($P < 0.001$), while the stat3-mus-1324 interference group showed a slightly weaker but still significant suppression of STAT3 expression ($P < 0.01$). In contrast, the STAT3 overexpression group displayed a significant increase in expression levels compared to the control group ($P < 0.05$).

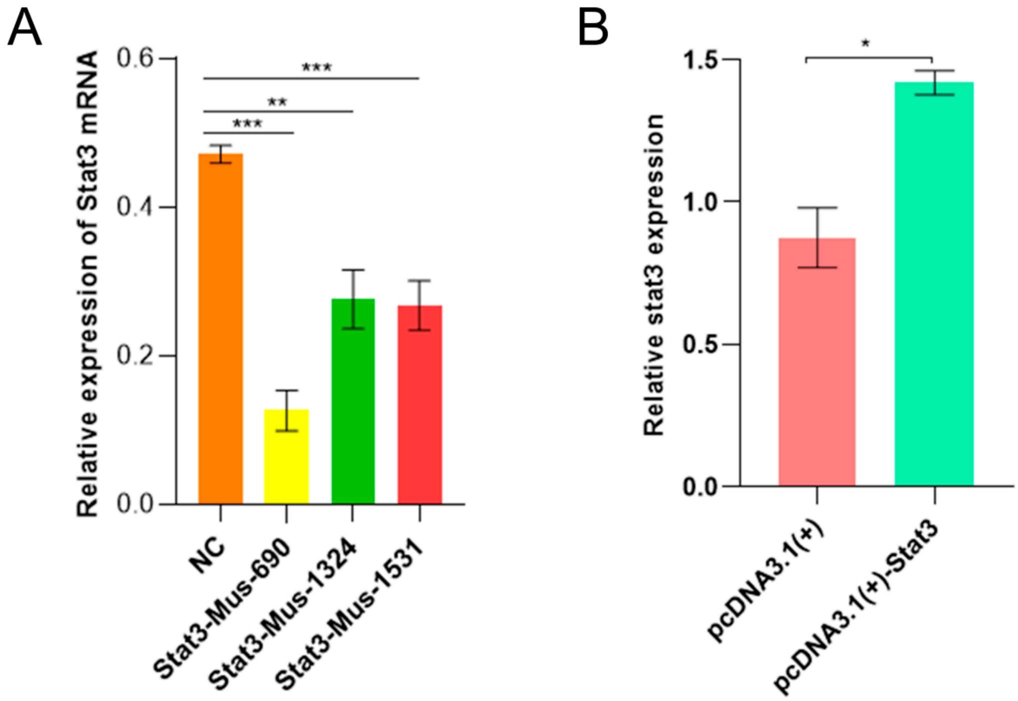

Validation of STAT3 knockdown/overexpression transfection efficiency in NCTC1469 cells. (A)Validation of STAT3 knockdown efficiency.Figure;(B)Validation of STAT3 overexpression efficiency.* P <0.05,** P <0.01,*** P <0.001
