# Supplementary material for: Single-Cell Transcriptome Reveals the Regulatory Role of STAT3 in Diquat-Induced Oxidative Stress in Piglet Hepatocytes
Source: Int J Mol Sci. 2025 Sep 19;26(18):9161. doi: 10.3390/ijms26189161 (PMC12471023; doi:10.3390/ijms26189161)
Supplement: Supplementary file 1 [file ijms-26-09161-s001.zip › Supplementary Material S2.pdf]

Measurement of SOD, MDA, and GSH in Liver Tissue

Sample preparation for SOD assay: Approximately 0.1 g of tissue (or 0.25 g for samples with high water content) was homogenized in 1 mL of extraction buffer on ice or at 4°C. The homogenate was centrifuged at 12,000 rpm for 10 min at 4°C, and the supernatant was collected as the test solution for subsequent analysis.

**Reagent composition** （μL）	**Sample tube**	**sample control tube**	**Blank tube 1** （Performed once only）	**Blank tube 2** （Performed once only）
Reagent 1	240	240	240	240
Reagent 2	60		60	
distilled water		60	60	120
Sample	60	60		
Reagent 3	60	60	60	60
Reagent 4	320	320	320	320

Mix thoroughly, incubate at room temperature for 30 min, and measure the absorbance (A) at 560 nm.

SOD activity (U/g fresh weight) = [Inhibition percentage ÷ (1 − Inhibition percentage) × V2] ÷ (W × V1 ÷ V) = 12.4 × Inhibition percentage ÷ (1 − Inhibition percentage) ÷ W × D

W — sample weight, g;D — sample dilution factor (1 for undiluted samples);

Sample preparation for MDA assay: Weigh approximately 0.1 g of tissue (for samples with high water content, up to 0.5 g may be used) and homogenize in 1 mL of extraction buffer using an ice bath. Centrifuge the homogenate at 12,000 rpm for 10 min at 4°C. Collect the supernatant and keep it on ice for subsequent analysis.

**composition** （μL）	**Test tube**
Working solution	300
Sample	200

After thorough mixing, the samples were incubated in a water bath at 90-95°C for 30 min, then immediately cooled on ice. Subsequently, centrifugation was performed at 25°C and 12,000 rpm for 10 min. A 200 μL aliquot of the supernatant was transferred to a 96-well plate, and the absorbance (A) was measured at 532 nm and 600 nm. The ΔA value was calculated as ΔA = A532- A600.

MDA content (nmol/g fresh weight) = $[\Delta A \div (\varepsilon \times d) \times V_2 \times 10^9] \div (W \times V_1 \div V) = 32.3 \times \Delta A \div W$.

W—sample weight, g;

GSH Tissue Sample Preparation: Approximately 0.1 g of tissue was weighed and homogenized in 1 mL of extraction buffer using an ice-cold mortar/pestle or homogenizer. The homogenate was centrifuged at 12,000 × g for 15 min at 4°C. The resulting supernatant was collected and maintained on ice for subsequent analysis.

**Reagent composition** （μL）	**Test tube**	**Control tube**

Sample	20	20
Reagent 1	120	160
Reagent 2	40	

Mix immediately and let stand for 5 min, then measure the absorbance (A) at 412 nm. The absorbance difference (ΔA) was calculated as ΔA = A test tube - A control tube.

GSH (μmol/g fresh weight) = $[(\Delta A + 0.0154) \div 1.0041 \times V_1] \div (W \times V_1 \div V) = 0.996 \times (\Delta A + 0.0154) \div W$

W — sample weight, g.
